# Apolipophorin-III Acts as a Positive Regulator of *Plasmodium* Development in *Anopheles stephensi*

**DOI:** 10.3389/fphys.2017.00185

**Published:** 2017-04-07

**Authors:** Rini Dhawan, Kuldeep Gupta, Mithilesh Kajla, Parik Kakani, Tania P. Choudhury, Sanjeev Kumar, Vikas Kumar, Lalita Gupta

**Affiliations:** ^1^Molecular Parasitology and Vector Biology Laboratory, Department of Biological Sciences, Birla Institute of Technology and SciencePilani, India; ^2^Department of Biotechnology, Chaudhary Bansi Lal UniversityBhiwani, India; ^3^Department of Zoology, Chaudhary Bansi Lal UniversityBhiwani, India

**Keywords:** *Anopheles stephensi*, Apolipophorin-III, immunity, *Plasmodium*, mosquito

## Abstract

Apolipophorin III (ApoLp-III) is a well-known hemolymph protein having a functional role in lipid transport and immune responses of insects. Here we report the molecular and functional characterization of *Anopheles stephensi* Apolipophorin-III (AsApoLp-III) gene. This gene consists of 679 nucleotides arranged into two exons of 45 and 540 bp that give an ORF encoding 194 amino acid residues. Excluding a putative signal peptide of the first 19 amino acid residues, the 175-residues in mature AsApoLp-III protein has a calculated molecular mass of 22 kDa. Phylogenetic analysis revealed the divergence of mosquitoes (Order Diptera) ApoLp-III from their counterparts in moths (Order: Lepidoptera). Also, it revealed a close relatedness of AsApoLp-III to ApoLp-III of *An. gambiae*. AsApoLp-III mRNA expression is strongly induced in *Plasmodium berghei* infected mosquito midguts suggesting its crucial role in parasite development. AsApoLp-III silencing decreased *P. berghei* oocysts numbers by 7.7 fold against controls. These effects might be due to the interruption of AsApoLp-III mediated lipid delivery to the developing oocysts. In addition, nitric oxide synthase (NOS), an antiplasmodial gene, is also highly induced in AsApoLp-III silenced midguts suggesting that this gene acts like an agonist and protects *Plasmodium* against the mosquito immunity.

## Introduction

Apolipophorin-III (ApoLp-III) is an amphipathic insect hemolymph protein that binds hydrophobically to lipoprotein surfaces and allows lipid transport in aqueous media (Van der Horst et al., 1993; Blacklock and Ryan, 1994). Homologous to a mammalian lipoprotein Apolipoprotein E, the insect ApoLp-III facilitates the transport of diacylglycerol (DAG) from fat body to flight muscles under the influence of adipokinetic hormones (Feingold et al., 1995). The remarkable lipid binding ability of ApoLp-III molecule is due to its structure, which is composed of five antiparallel amphipathic helices forming a bundle arranged in an up-and-down topology. The hydrophobic regions of the helices are directed into the interior whereas the hydrophilic regions are presented on the surface of the bundle, preventing precipitation of ApoLp-III in the aqueous environment of hemolymph. Upon lipid interaction, the ApoLp-III molecule undergoes conformational changes that lead to the opening of the bundle of the helices (Breiter et al., 1991; Weers and Ryan, 2006). Therefore, the non-lipid associated globular form and the lipid associated form are the two conformational states of this protein (Dettloff et al., 2001; Park et al., 2005).

Recently, insect ApoLp-III was documented as an immune-stimulating protein mediating both humoral and cellular immune responses (Kim et al., 2004; Whitten et al., 2004; Song et al., 2008; Zdybicka-Barabas and Cytryńska, 2011). ApoLp-III binds to a varied range of immune elicitors including lipoteichoic acid (LTA), present in cell wall of Gram-positive bacteria (Halwani and Dunphy, 1999; Halwani et al., 2001), lipopolysaccharide (LPS) layer of Gram-negative bacteria (Kato et al., 1994; Dunphy and Halwani, 1997), and beta 1, 3 glucan of fungal cell wall (Whitten et al., 2004). These binding properties impart ApoLp-III to have a role pertaining to the pathogen recognition receptor (PRR). It is also known to stimulate encapsulation, a hemolymph immune reaction that occurs in response to the systemic introduction of foreign, non-self substances that are too large to be phagocytized by hemocytes (Zdybicka-Barabas and Cytrynska, 2013). Taken together, ApoLp-III lucratively establishes itself as a multifunctional hemolymph protein involved in both humoral and cellular immune responses in several species of insects, such as *Heliothis virescens* (Chung and Ourth, 2002), *Hyphantria cunea* (Kim et al., 2004), *Anopheles gambiae* (Gupta et al., 2010), *Thitarodes pui* (Sun et al., 2012), and *Tribolium castaneum* (Contreras et al., 2013).

Previous studies revealed that the silencing of ApoLp-III gene greatly enhanced *Plasmodium* infection in *An. gambiae* (G3) mosquito (Gupta et al., 2010). However, it has no effect on parasite development in *An. gambiae* Yaounde' strain (Mendes et al., 2008). These reports revealed two different outcomes of anopheline ApoLp-III in regulation of *Plasmodium* development. Thus, we designed the present study to characterize ApoLp-III gene in a major Indian malaria vector *An. stephensi* and understanding its role in regulation of *Plasmodium* development using RNA interference (RNAi). We found that ApoLp-III silencing significantly reduced the number of *P. berghei* oocysts in *An. stephensi* and thus, acts like a positive regulator of malaria parasite development. The contrasting behavior of this gene in Indian species also shed light on the vast range of immune responses that exist between the specific strains of mosquito and parasite.

## Materials and methods

### Mosquito rearing and *Plasmodium* infection

*An. stephensi* larvae were fed on a 1:1 mixture of dog food (PetLover‘s crunch milkbiscuit, India) and fish food (Gold Tokyo, India). Adult mosquitoes were allowed to feed on cotton soaked in 10% sugar solution ad-libitum and blood fed on anesthetized mice (Swiss albino) for colony propagation. They were maintained in a 12:12 h light: Dark cycle at 28°C and 80% relative humidity as before (Dhawan et al., 2015; Kajla et al., 2016a). Female mosquitoes were infected after feeding them on *P. berghei*-infected mice. Parasitemia in mice was analyzed and potential infectivity to mosquitoes was determined through exflagellation assays as previously described (Billker et al., 1997). In all experiments, mosquitoes were infected with mice having two to three exflagellations/field under 40X objective. Blood-fed mosquitoes were kept at 21°C and 80% humidity, the permissive temperature for parasite development as discussed before (Gupta et al., 2009; Kumar et al., 2010).

### Identification, sequence retrieval, and *in-silico* analysis of *An*. *stephensi* ApoLp-III

The *An. gambiae* ApoLp-III cDNA sequence was selected as query to identify a putative AsApoLp-III cDNA from the *Anopheles stephensi* genome database using the NCBI basic local alignment search tool (BLAST). Putative AsApoLp-III from the available contig (No: KE388890.1) was identified. To confirm the putative AsApoLp-III sequence, specific primers were designed to amplify, clone and sequenced using *An. stephensi* cDNA as template. The primers AsApoLp-III Fwd: 5′-AGCCCAATTTCTTCCAGACC-3′ and AsApoLpIII Rev: 5′-CGGTTGCTTCAGCTCGTT-3′ were used to amplify 482 bp cDNA fragment. The amplified product was cloned into PCR-TOPO TA cloning vector (Invitrogen) and sequenced. This clone was further used for dsRNA synthesis. The cloned AsApoLp-III cDNA sequence was also deployed to find out the genomic structure of AsApoLp-III by using the BLASTN program. The ORF was predicted using GenScan (http://genes.mit.edu/GENSCAN.html) (Burge and Karlin, 1997) and the functional domains in the protein were predicted using NCBI conserved domain search tool.

SignalP software (Petersen et al., 2011) was deployed to find out the signal sequence in AsApoLp-III protein. Putative phosphorylation or glycosylation sites were identified in this protein using NetPhos 2.0 and NetNGly1.0 server, respectively at http://www.cbs.dtu.dk. Prediction of protein tertiary structure was carried through Phyre2 server (Kelly and Sternberg, 2009) and ligand binding site were identified using 3DLigand site prediction server (Wass et al., 2010). Protein sequences of ApoLp-IIIs from other insect species were retrieved from NCBI to align them with AsApoLp-III by Clustal Omega and phylogenetic tree was constructed by the neighbor-joining method using the MEGA program (version 5.0) with bootstrap value of 1,000 replicates (Tamura et al., 2011).

### Sample collection

Midguts were dissected from uninfected (control) or *P. berghei*-infected mice blood fed mosquitoes at 0, 3, 6, 12, 18, and 24 h post-feeding. The carcasses (rest of the body except midgut) were also collected in parallel at the same time points. All the samples were kept in RNAlater solution (Qiagen) and stored at −80°C.

### RNA isolation, cDNA synthesis, and RT-PCR

Total RNA from the above-collected samples was isolated using RNeasy Mini Kit (Qiagen) following manufacturer's instructions. First-strand cDNA was synthesized from total RNA using QuantiTect reverse transcription kit (Qiagen). Expression profile of different genes was carried through semi-quantitative real time PCR using SYBR green supermix in an IQ5 multicolor real-time PCR detection system (Bio-Rad) where ribosomal protein subunit S7 mRNA was used as internal loading control as described before (Salazar et al., 1993; Kajla et al., 2016b). Primer pairs used for different genes were following, S7-Fwd: 5′-GGCGATCATCATCTACGT-3′ and S7-Rev: 5′-GTAGCTGCTGCAAACTTCGG-3′, AsApoLp-III Fwd: 5′-AGCCCAATTTCTTCCAGACC-3′, and AsApoLpIII Rev: 5′-CGGTTGCTTCAGCTCGTT-3′. PCR cycle parameters involved an initial denaturation at 95°C for 5 min, 40 cycles of 10 s at 94°C, 20 s at 58°C, and 30 s at 72°C. Fluorescence readings were taken at 75°C after each cycle. A final extension at 72°C for 10 min was completed before deriving a melting curve, to confirm the identity of PCR products. Each experiment was performed in three independent biological replicates. Fold values were calculated using ^ΔΔ^Ct method (Livak and Schmittgen, 2001; Dhawan et al., 2015) and statistical significance of the data was analyzed by Student's *t-*test.

### dsRNA synthesis

A 218-bp fragment of the lacZ gene was amplified using the primers (5′ to 3′) Fwd-GAGTCAGTGAGCGAGGAAGC and Rev-TATCCGCTCACAATTCCACA and cloned into the PCR-TOPO TA vector. For AsApoLp-III, the 482-bp clone, as mentioned above, was used for dsRNA synthesis. This recombinant plasmid already has a T7 promoter side at one end however; another T7 promoter side at other end of the fragment was incorporated by amplifying the cloned insert using the following primers: M13F-GTAAAACGACGGCCAGT and T7-M13R-CTCGAGTAATACGACTCACTATAGGGCAGGAAACAGCTATGAC. The cloned plasmid served as template to synthesize dsRNA using MEGAscript RNAi kit (Cat No. AM1626, Ambion, Austin, TX). dsRNA was purified using a Microcon YM-100 filter (Millipore) and concentrated to 3 μg/μl in water.

### AsApoLp-III silencing in female mosquitoes

For gene silencing, 69 nl of 3 μg/μl AsApoLp-III dsRNA was injected into 2 days old female mosquitoes. Control mosquitoes were injected with same amount of dsLacZ. After 4 days post-injection these females were fed on a transgenic GFP-expressing *P. berghei* infected mouse and maintained at 21°C in an incubator as mentioned in Materials and Methods. After 7 days the mosquito midguts were dissected and fixed in 4% formaldehyde as before (Kumar et al., 2004; Gupta et al., 2005). The numbers of developing oocysts were counted in these midguts with the help of a fluorescent microscope (Olympus). The distribution of the number of oocysts in control and silenced midguts were compared using the Kolmogorov-Smirnov (KS) test. The percentage silencing of the AsApoLp-III gene in control and silenced midguts was also analyzed using AsApoSil-Fwd: 5′-GCGAAGCTTCTGTGCCTTAT-3′ and AsApoSil-Rev: 5′-CTCGAATGCCACCTCAATCT-3′ primers.

### Effect of AsApoLp-III silencing on NOS expression during *P. berghei* infection

To analyze the effect of AsApoLp-III silencing on NOS expression, the females with reduced endogenous level of AsApoLp-III mRNA or sham treated controls were fed on transgenic GFP-*P. berghei* infected mice as mentioned above. Mosquito midguts were dissected 24 h post-infection, which is the time when ookinetes invade the mosquito midgut epithelium (Kumar et al., 2004, 2010). The expression levels of NOS gene were analyzed in these midguts using following primers, NOS-Fwd: 5′-ACATCAAGACGGAAATGGTTG-3′ and NOS-Rev: 5′- ACAGACGTAGATGTGGGCCTT-3′.

## Results and discussion

### Identification and *in silico* analysis of AsApoLp-III gene

**Figure 1:**
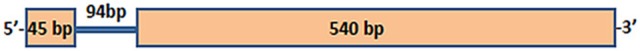
**Genomic organization of AsApoLp-III gene**. AsApoLp-III gene has two exons (45 bp and 540 bp) that are separated by a 94 bp intron.

**Figure 2:**
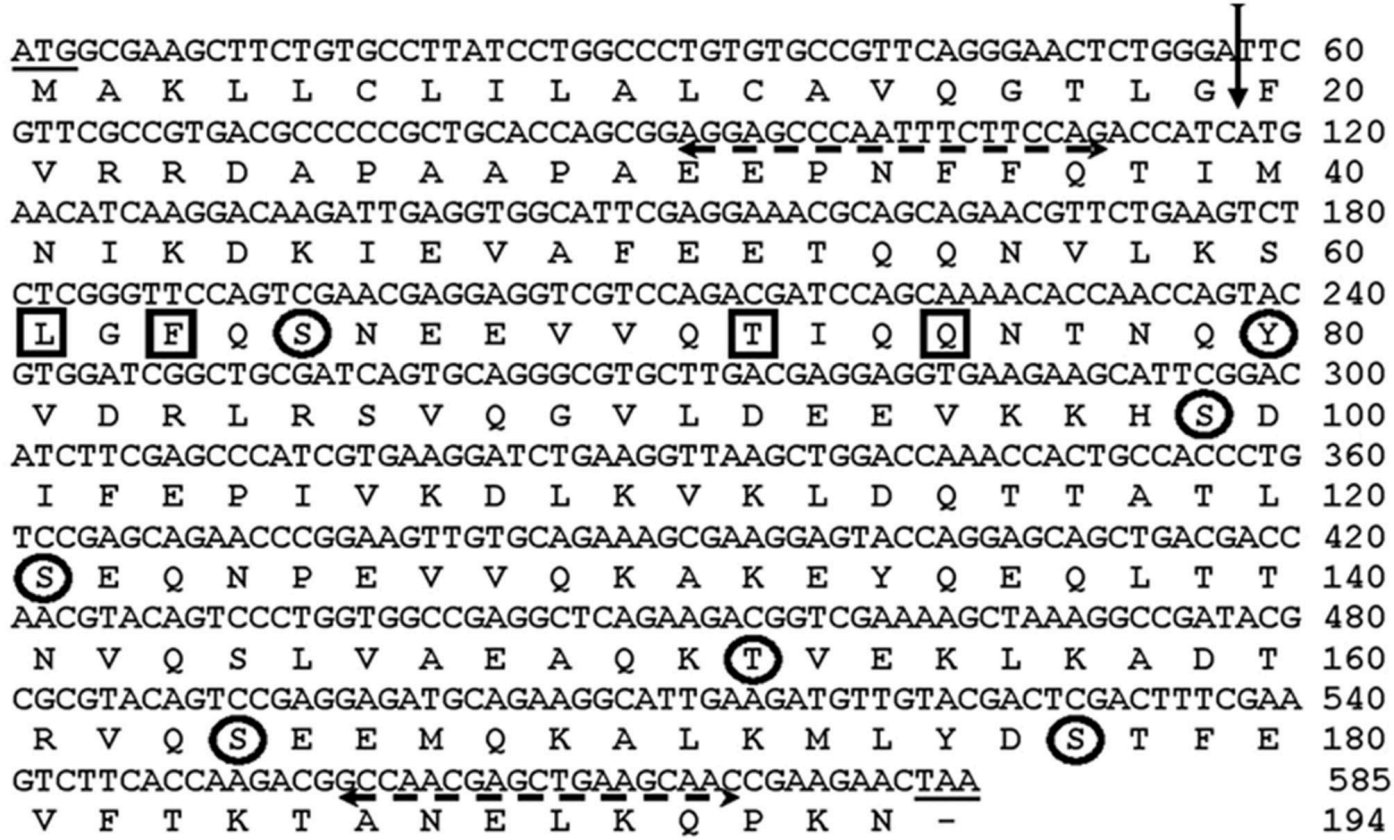
**Nucleotide and deduced amino acid sequence of AsApoLp-III gene**. The vertical arrow indicates signal peptidase cleavage site in the deduced polypeptide between the amino acid 19 and 20 (TLG_19_-F_20_V). Primer sequences are shown with dashed horizontal arrows. The encircled amino acids indicate the predicted phosphorylation sites (Ser, Thr, Tyr). The interacting sites of the protein are shown by the boxed residues.

In an attempt toward identification and functional characterization of immune-related genes, we retrieved ApoLp-III nucleotide sequence from *An. stephensi* genome database using *An. gambiae* ApoLp-III as the reference. Based on the conserved regions of *An. gambiae* ApoLp-III and Genscan program we determined the putative AsApoLp-III gene. It contains 679 nucleotides with 45 bp and 540 bp exons that are separated by a 94 bp intron (Figure 1). This arrangement is in conformity to the AgApoLp-III gene, which has 815 bp long CDS arranged into 173 bp and 642 bp exons that are separated by a 89 bp intron (Gupta et al., 2010). Conclusively, a 585 bp coding region of AsApoLp-III gene encodes 194 amino acid long protein (Figure 2). The BLASTp results for putative AsApoLp-III protein revealed >80% identity with AgApoLp-III. The deduced polypeptide when subjected to domain analysis using NCBI CD search tool revealed the presence of domain from Apolipophorin-III superfamily.

AsApoLp-III sequence analysis using SignalP (http://www.cbs.dtu.dk/services/SignalP) predicted that this is a secreted protein, having a signal peptide at the N terminal with the cleavage site between 19 and 20^th^ (TLG_19_-F_20_V) amino acid residues (Figure 2). The cleavage of signal peptide results in the generation of a mature 175 amino acids long AsApoLp-III protein with a calculated molecular mass of 22 kDa and theoretical pI of 5.13. These parameters of AsApoLp-III protein are comparable with previously identified insect ApoLp-IIIs (Weers and Ryan, 2006). The predicted signal peptide of 19 amino acids in the N-terminal region may transit the polypeptide into the secretary pathway where the hemolymph protein can exist in both lipid-free and lipophorin-bound forms.

In general, the post-translational modifications (PTM), through the covalent addition of functional groups or proteolytic cleavage of regulatory subunits, alter the functional diversity of proteins. These modifications influence almost all aspects of normal cell biology and pathogenesis. Therefore, we analyzed phosphorylation and glycosylation post-translational modification sites in the predicted AsApoLp-III protein using NetPhos 2.0 and NetNGly1.0 server, respectively at http://www.cbs.dtu.dk. It was observed that the polypeptide lacks any putative glycosylation site but comprises 7 putative Ser/Thr and Tyr residues that may undergo phosphorylation (Figure 2). Though, glycosylation has been observed in many ApoLp-IIIs from orthoptera (Chino and Yazawa, 1986) however, there are certain examples that belong to non-glycosylated ApoLp-III also as reported earlier (Son and Kim, 2011). The active site of the ApoLp-III protein consists of Leu-61, Phe-63, Thr-72, and Gln-75 as predicted by the 3D Ligand Site software (Figure 2).

**Figure 3:**
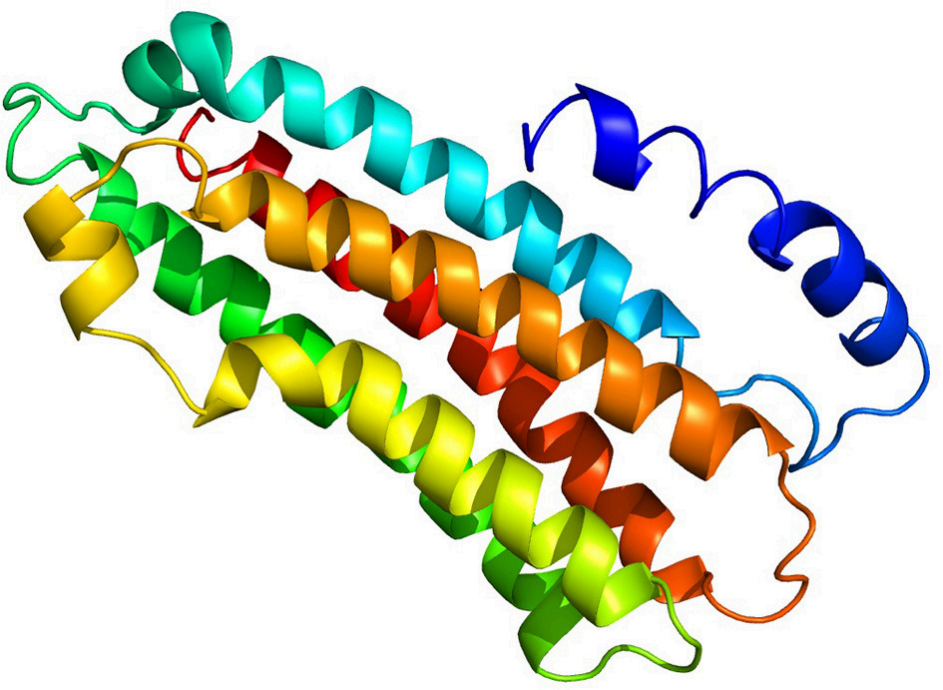
**Predicted 3D structure of putative AsApoLp-III protein**. The AsApoLp-III protein consists of five long alpha-helices connected through short loops. The predicted model was constructed based on the *M. sexta* ApoLp-III as the template [PDB id: 1eq1]. Protein structures were predicted using Phyre2 server.

Tertiary structure of the predicted AsApoLp-III protein was generated by Phyre2 program as before (Kelly and Sternberg, 2009). Of all, 89% of its total residues were modeled with 99.7% confidence against *Manduca sexta* ApoLp-III as reference (PDB ID: 1EQ1) (Figure 3). It is observed that despite the disparate similarity in the primary structure of ApoLp-III protein, ranging from >70% with dipterans to as low as 19% with lepidopteran, there seem to be a firm conservation in the tertiary structure of the protein (Gupta et al., 2010). These findings indicated that the functional conservation of this protein might have a minor selection pressure. The Ramchandran Plot analysis predicted that 98% residues of the proposed model of AsApoLp-III lie in favorable region rendering the reliability of this model.

**Figure 4:**
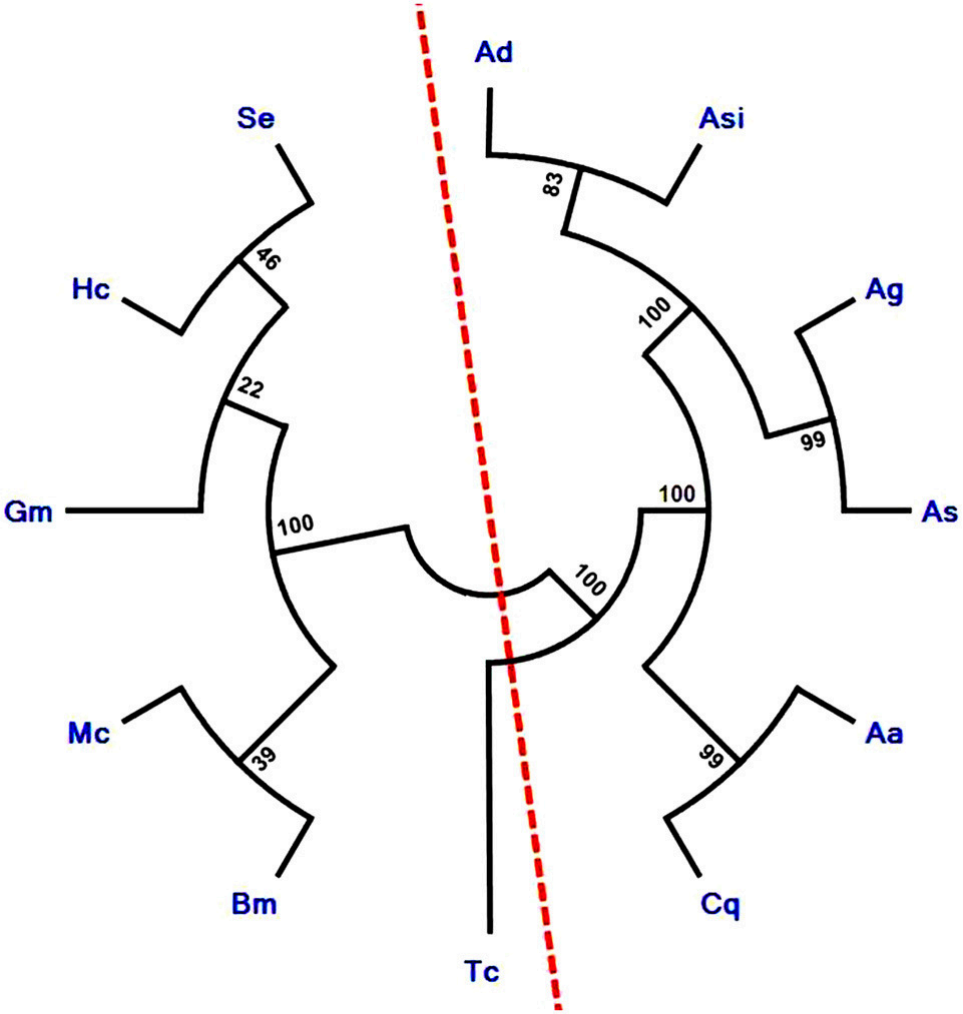
**Phylogenetic analysis of insect ApoLp-III family proteins**. The phylogenetic tree was constructed using Neighbor-joining algorithm. Sequences used for phylogenetic analysis are Ag, *Anopheles gambiae* (XP_003436408); Ad, *Anopheles darlingi* (ETN60814); Asi, *Anopheles sinensis* (ADN52300); Cq, *Culex quinquefasciatus* (XP_001849278); Aa, *Aedes aegypti* (XP_001659524); As, *Anopheles stephensi* (KU051523); Se, *Spodoptera exigua* (AEW24424.1); Gm, *Galleria mellonella* (CAA07363.1); Mc, *Manduca sexta* (P13276); Bm, *Bombyx mori* (AAQ17038.1); Hc, *Hyphantria cunea* (AAQ24031); Tc, *Tribolium casteneum* (EFA05722.1). The analysis revealed clear divergence of ApoLp-III of mosquito species from moths as indicated by the red color imaginary line. The numbers on the branches represent the % of 1000 bootstrap.

Phylogenetic relationship among several lepidopteran and dipterans ApoLp-III proteins, divide them into two separate groups. It is noteworthy to mention that AsApoLp-III is exclusively grouped with mosquito ApoLp-III proteins. In addition, AsApoLp-III shares close relatedness with *An. gambiae* ApoLp-III (Figure 4). The reason of the clear divergence of this gene from their lepidopteran counterparts can be attributed to the adaptive differences in biology and feeding behavior seen within the insect orders.

### Cloning and sequence analysis

After the retrieval of ApoLp-III gene in the contig (No: KE388890.1) of *An. stephensi* genome, the sequence was confirmed through cloning and sequencing as discussed before. BLAST searches confirmed that AsApoLp-III gene shares more than 80% similarity with mosquito ApoLp-III genes and also exhibits a considerable sequence resemblance with Apolipophorins of other insects. This is the foremost report of Apolipophorin-III from *An. stephensi* and its gene sequence has been submitted to NCBI GenBank database (Accession No: KU051523).

### Expression kinetics of AsApoLp-III gene in response to *Plasmodium* infection

**Figure 5:**
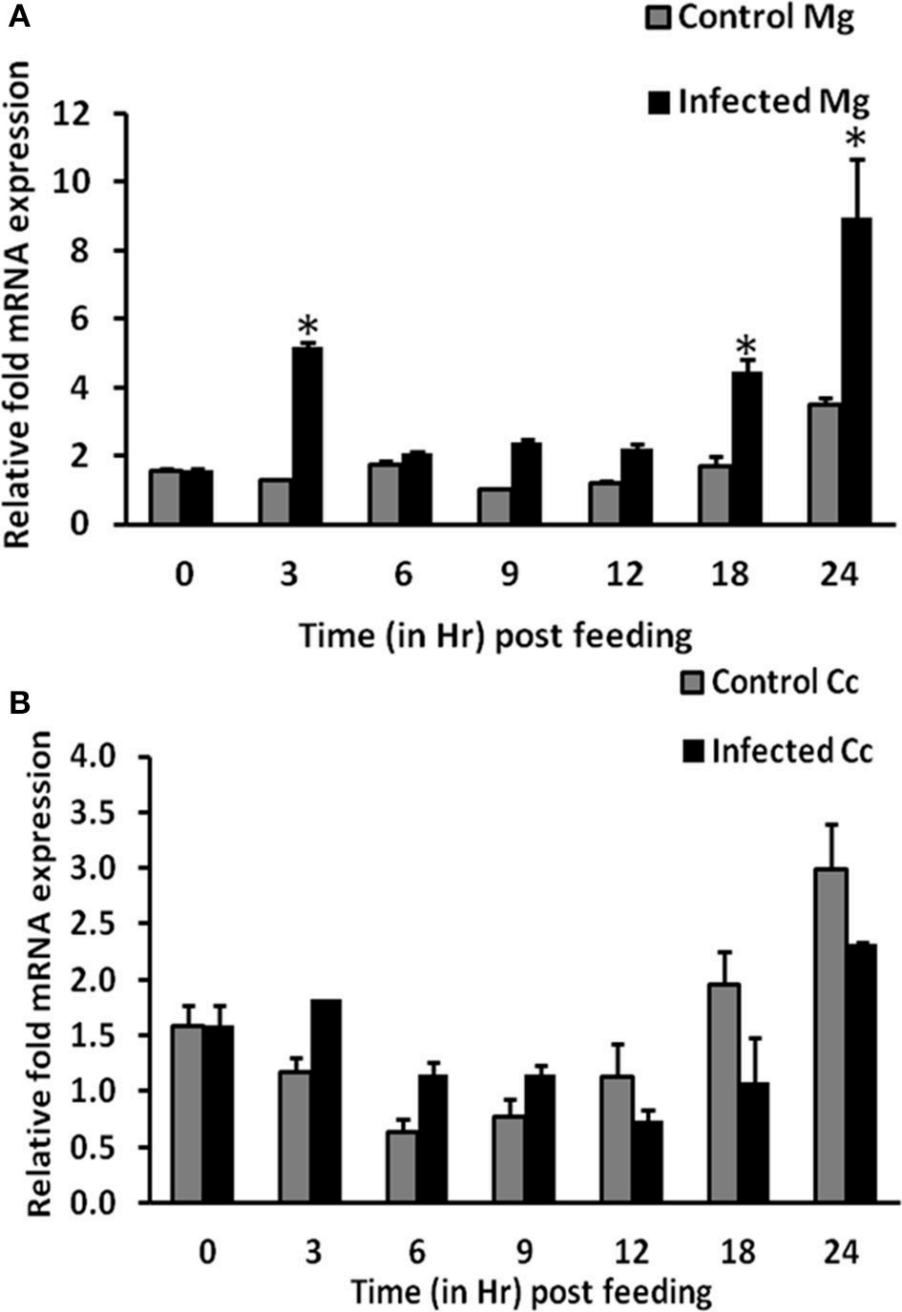
**Time kinetics of AsApoLp-III gene expression in *Plasmodium berghei* infected females. (A)** Relative AsApoLp-III mRNA levels in female midguts fed on an uninfected (control) or *P. berghei*-infected mouse. Samples were collected at different time points after the blood feeding. **(B)** Same as **(A)** but carcass samples were collected from the same mosquitoes. Ribosomal S7 protein mRNA levels were used as internal loading reference. The asterisk denoted statistically significant difference in relative mRNA levels of control and infected samples.

To find out the role of AsApoLp-III in immunity, AsApoLp-III mRNA expression in the adult female midgut after *P. berghei* infected blood feeding at varied time points was investigated by qRT-PCR. As shown in Figure 5A, AsApoLp-III mRNA expression in midguts was significantly upregulated (~4 times) at 3 h after infected blood feeding compared to the controls. This time point represents *Plasmodium* pre-ookinete stage when fertilization and zygote formation takes place in the gut lumen of infected mosquitoes. Following up-regulation at 3 h, a gradual reduction of AsApoLp-III transcript levels was observed till 12 h of post-infection. Furthermore, the AsApoLp-III transcript levels increased significantly at 18 and 24 h post-infection against controls (Figure 5A). The significance of 24 h time point corresponds to ookinetes invasion of the midgut epithelium (Kumar et al., 2004; Gupta et al., 2010).

Transcriptional regulation of a mosquito gene indicates that it might have a crucial role against the stressful situation of parasite infection. The time kinetics of AsApoLp-III expression in *P. berghei* infected midguts revealed a biphasic pattern of induction (Figure 5A). Induction pattern at 24 h after *P. berghei* infection is in accordance with *An. gambiae* expression pattern (Gupta et al., 2010). In *An. gambiae*, it was observed that AgApoLp-III gene was induced due to the midgut epithelium invasion by the ookinetes, as mosquitoes fed on an infected mouse and maintained at 28°C, a non-permissible temperature for ookinete development, showed no induction of ApoLp-III gene (Gupta et al., 2010). The up-regulation of AsApoLp-III gene at critical time points of 3 and 24 h post-infection might indicates its decisive role in establishment of *Plasmodium* infection.

The expression kinetics of AsApoLp-III gene was also profiled for carcasses (mosquito body except midgut) through qRT-PCR. In this body compartment, the expression of AsApoLp-III gene is indifferent between control and infected mosquitoes at all the time points post-blood feeding (Figure 5B). In contrary to midgut expression, AsApoLp-III demonstrates infection independent temporal induction in the carcass, which is in agreement with the notable abundance of apolipoproteins in the fat body. Moreover, AsApoLp-III induction pattern in *P. berghei* infected carcasses revealed that it is expressed more in controls than the infected samples at crucial time point of ookinete invasion. This indicated that AsApoLp-III gene might be more effective as immune molecule in the midgut tissue, which is the first immune organ encountered by the parasite.

### Effect of AsApoLp-III silencing on *Plasmodium* infection

**Figure 6:**
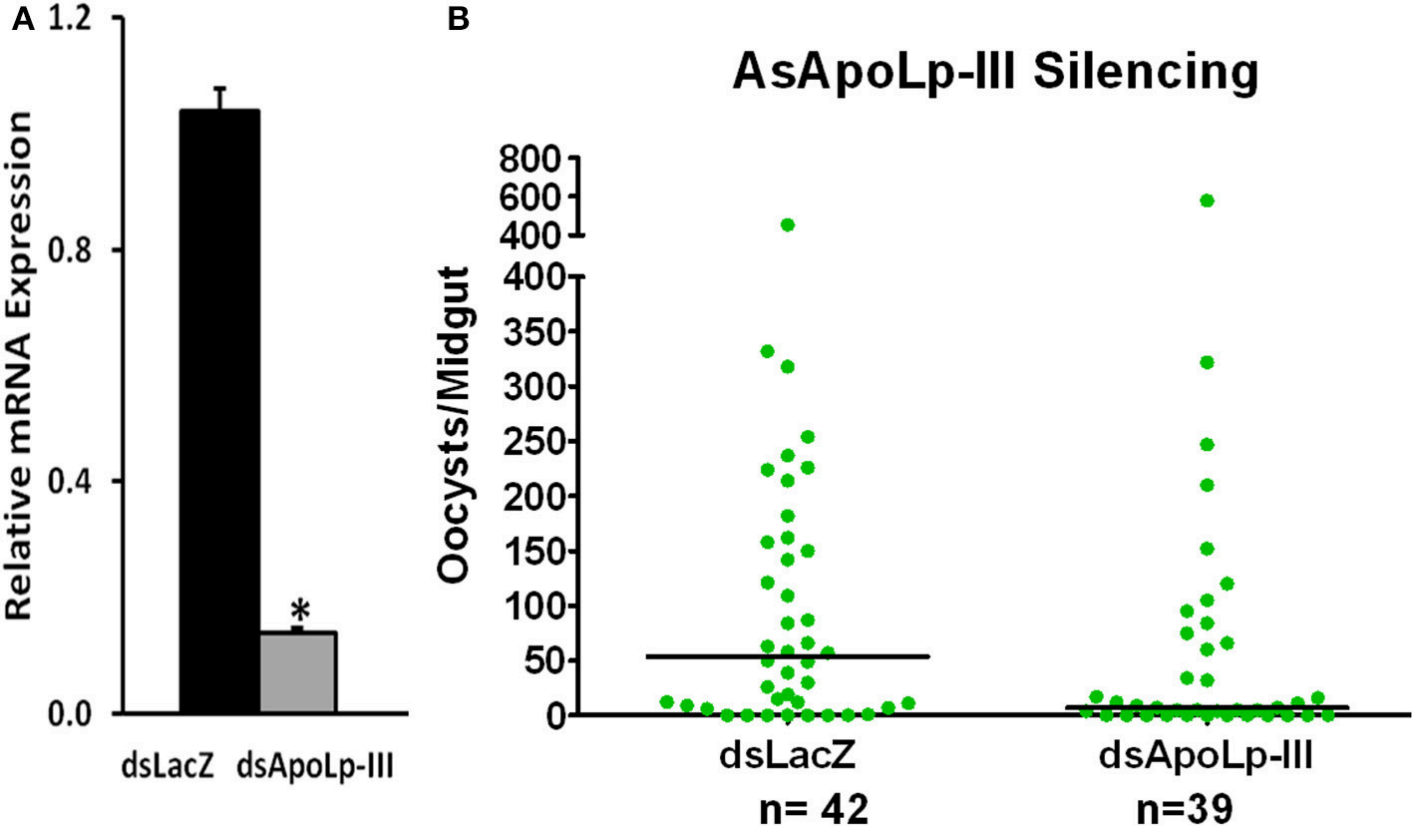
**Effect of AsApoLp-III silencing on *P. berghei* infection in *An. stephensi* females. (A)** Relative abundance of midgut AsApoLp-III mRNA in *An. stephensi* mosquitoes that were injected with AsApoLp-III (silenced) or LacZ (control) dsRNA. **(B)** Effect of AsApoLp-III silencing on the number of live oocysts (green dots) in midguts analyzed 7 days post-infection. Dots represent the number of parasites present in individual midgut, and the median number of parasites is indicated by the horizontal line. Distributions are compared using the Kolmogorov-Smirnov test; *n*, number of mosquitoes. The statistically significant difference in relative mRNA levels is denoted by an asterisk.

AsApoLp-III gene was silenced through RNAi to evaluate its effect on *Plasmodium* development. The silencing reduced the level of endogenous AsApoLp-III mRNA by 85% when compared to the controls (Figure 6A). Furthermore, AsApoLp-III silencing decreased the median number of live oocysts by 7 fold against controls (*P* < 0.0001) (Figure 6B) indicating that this gene is required for effective infection and therefore, acts as *Plasmodium* agonist. We propose that AsApoLp-III might be transporting the lipids from hemolymph to the developing oocysts and in its absence this stage of parasite becomes more susceptible to the immune attack and subsequently undergoes degeneration. These assumptions are supported by the previous study where developing *Plasmodium* oocysts are reported to be dependent on mosquito lipids (Atella et al., 2009).

**Figure 7:**
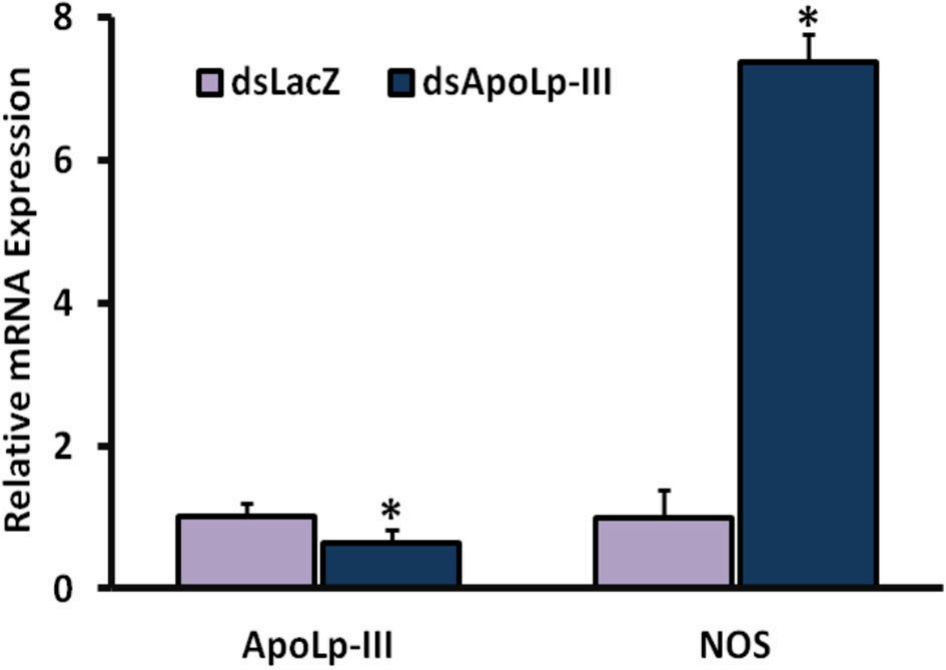
**Effect of AsApoLp-III silencing on NOS expression**. The expression of NOS gene was compared in the midguts of control or AsApoLp-III silenced mosquitoes at 24 h post *Plasmodium* infection when ookinetes invade the midgut epithelium. Relative levels of AsApoLp-III in these samples revealed the significant reduction of endogenous mRNA as a result of RNA interference. The statistically significant difference in relative mRNA levels is denoted by an asterisk.

Interestingly, a seven fold induced expression of nitric oxide synthase (NOS) gene was also observed in AsApoLp-III silenced midguts after 24 h post *Plasmodium* infection when compared to the sham treated controls (Figure 7). In fact, NOS is a well-documented antiplasmodial molecule and the ookinetes must migrate out of the midgut cell before they are damaged by the action of this protein (Han and Barillas-Mury, 2002; Kumar et al., 2004, 2010; Gupta et al., 2005; Kajla et al., 2017). Thus, it might be possible that the early event of epithelial invasion by the ookinetes is also negatively affected in AsApoLp-III silenced midguts due to the toxic effect of NOS. This fact is evident from an earlier study where ApoI/II protein is reported to facilitate the movement of ookinetes through *An. gambiae* (Yaounde' strain) midgut epithelium (Mendes et al., 2008). This study suggested that massive droplets appeared at basal side of the midgut cells by the ApoI/II mediated lipid mobilization guides the ookinetes to exit the invaded cell. In ApoI/II silenced midguts, the ookinetes are trapped in the toxic environment of the invaded cell and fail to develop into the oocysts (Billingsley and Lehane, 1996; Mendes et al., 2008). We believe that *An. stephensi* ApoLp-III might be also displaying a similar effect during ookinete migration through the midgut epithelium. The induced expression of ApoLp-III gene in *Plasmodium* infected *An. stephensi* midguts (Figure 5A of this study) and the presence of ApoLp-III protein in *An. gambiae* (G3) midgut epithelial cells (Gupta et al., 2010) revealed the association of this protein with the invasive ookinetes and might further support this concept.

In the view of the above discussion, AsApoLp-III seems to be facilitating the ookinetes invasion of the midgut epithelium, successful development of oocysts and their protection against host immunity. Further studies are demanded to understand the details of these mechanisms through which ookinete migration and development is supported by lipoproteins.

## Conclusion

India contributes 70% of malaria cases and 69% of malaria deaths in the South-East Asia region. *An. stephensi* is one of the major vectors responsible for malaria transmission in this region. Transmission blocking with the utilization of vector immunity is the most appealing approach as *Plasmodium* undergoes an important bottleneck situation during its development in the mosquito host. Molecular interactions between mosquitoes and malaria parasites studied in the laboratory models identified several mosquito immune-related genes, which affect the resulting infection in either positive or negative manner.

Here, we have discussed the positive regulatory effect of Apolipophorin-III (ApoLp-III) gene in *An. stephensi* that plays a decisive role in the survival of *P. berghei*. Reduced endogenous expression of AsApoLp-III leads to the decreased development of oocysts. Thus, we propose that AsApoLp-III protein might facilitate an easy egress of the parasite through midgut epithelium and/or further development into the oocysts. This view is further strengthened with the increased level of NOS mRNA at 24 h post-infection in the ApoLp-III silenced mosquitoes. It can be conclusively said that in the absence of ApoLp-III *Plasmodium* are trapped in the toxic environment of *An. stephensi* midgut, which eventually leads to lower infectivity.

However, the decreased numbers of oocysts at 7 days post-infection in the midgut of *An. stephensi* is in contrast to the results obtained on silencing of AgApoLp-III in susceptible G3 and refractory L3-5 mosquitoes that showed increased number of ookinetes (Gupta et al., 2010) whereas silencing of ApoLp-III in *An. gambiae* Yaounde' strain females showed no effect on parasite development (Mendes et al., 2008). This contrasting behavior of the gene in different species as well as different strains of same species put in forth the view that there occurs a huge assortment of compatibility, defined by the degree to which the immune system restricts the infection between particular strains of mosquitoes and specific parasites strains.

The real mechanism by which ApoLp-III functions in *An. stephensi* is yet to be explored. Experiments conducted in this manuscript indicated that this gene is playing an influential role in *Plasmodium* development. Categorically, this is the first report that indicates the involvement of ApoLp-III gene in Indian susceptible strain *An. stephensi*, which is responsible for major malarial transmission in the country.

## Ethics statement

This study was carried out in accordance to the protocol IAEC/RES/18/01 that was approved by the Institutional Animal Ethical Committee at BITS-Pilani.

## Author contributions

RD, SK, and LG designed the experiments. RD, KG, MK, TC, PK, and VK collected samples and performed experiments. RD, KG, LG, and SK performed ApoLp-III silencing experiments. KG, RD, SK, and LG analyzed the data and wrote the manuscript with input from all authors. All the authors read and approved the manuscript.

### Conflict of interest statement

The authors declare that the research was conducted in the absence of any commercial or financial relationships that could be construed as a potential conflict of interest. The reviewer JS and handling Editor declared their shared affiliation, and the handling Editor states that the process nevertheless met the standards of a fair and objective review.
